# Book Reviews

**Published:** 1808-12

**Authors:** 


### CRITICAL ANALYSIS

OF THE

### RECENT PUBLICATIONS

ON THE

### DIFFERENT BRANCHES OF PHYSIC, SURGERY, AND MEDICAL PHILOSOPHY.

---

*Cases of Diabetes, Consumption, &c. With Observations on the History and Treatment of Disease in General.* By ROBERT WATT, Member of the Faculty of Physicians and Surgeons, Glasgow. 8vo. London. 1808.

THESE Cases are preceded by an Introduction, in which we are told that the practice has been long prevalent in the North, of too indiscriminate feeding, and the apprehensions of blood-letting much too great. We have reason to believe the author is right, by the general sentiment which was excited on the first appearance of Dr. Hamilton's book, in favour of another evacuation. A further remark ows, which we should have thought less necessary, in favour of mercury. "This sentiment," says our author, "is not in unison with the anathemas of some modern declaimers, but is consonant to the experience of those who have employed this valuable medicine to eradicate, not to palliate, disease."

If by this our author means to object against the mode of *trying* mercury, in the small way, as an experiment, no one can object to it more than ourselves; nor can any impartial practitioner refuse to admit, that he has found many and great inconveniences resulting from this empirical practice. For the same reason, it would be absurd to lose sight of so powerful a medicine in many formidable diseases, or to use, without ascertaining its full effects.

The first case related of diabetes is of a labourer, 36 years of age, originally of a very robust constitution, and accustomed to hard work, who had a constitutional or habitual aversion to animal food. This, however, he endeavoured to conquer. It could not be ascertained how far he succeeded, but he died four months after his application for relief.

Some observations follow on this case. First, that Dr. Heberden does not seem founded in his remark, that Diabetes is a disease of debility, and for the most part only a prelude to approaching dissolution in the aged or infirm. The present, and several other subjects witnessed by our author, were of the middle age, and of an athletic figure and constitution. Another remark he adverts to, from the same author, that the urine is rarely sweet, whilst Dr. Cullen asserts that this may be considered as the chief diagnostic of the disease. Each of these physicians, venerable for their years, their extensive practice, and accurate remarks, had seen about the same number of cases. The author very candidly accounts for their difference of opinion.

"I have always thought," says he, "that the division of Diabetes into Mellitus and Insipidus, was more fanciful than real, and more systematic than useful. That in one instance the urine is sweet, and in another insipid, is evident enough; but unless we can say where the one disease terminates, and where the other begins, or unless it suggest some variety in the practice, the distinction serves only to perplex the practitioner.

"The fact seems to be, that wherever there is a permanently increased, and altered secretion of urine, if it be carefully analysed, less or more saccharine matter will be found; but, if it be merely tried by the taste, in perhaps more than one half of the cases that occur, it would be pronounced insipid. The variety then is only in degree, and may perhaps be totally disregarded in practice. Dr. Bardsley has very properly remarked, that "when the sensible qualities of this fluid did not point out the least saccharine impregnation, yet, on exposing an extract, obtained from it by evaporation, to the test of chemical analysis, it was found to contain more or less of the oxalic acid." The saccharine matter may be present, but so covered with other substances, that it cannot be at all detected by the taste.

"These observations seem to account for the remarkable difference of opinion entertained by the two authors. Cullen probably drew his inference from the chemical analysis of diabetic urine, Heberden from simply tasting it."

The second subject was similar to the former in his employments, his habits, and his age. The disease was well marked, and long continued. The usual remedies proving ineffectual, the author was induced, by the appearance of a slight hæmoptoe, to try the effect of blood-letting: his success, though at first small, was sufficient to authorise a perseverance in so generally fatal a complaint.

"In this case," remarks our author, "venesection was employed in the most forbidding circumstances. The pulse was slow, feeble, and not altogether regular—his strength and spirits were almost gone—the lower extremities had been œdematous to the haunches, and were always cold and lifeless. When newly drawn, the blood was extremely dark; on cooling, the crassamentum was found to be as black as pitch, and totally devoid of tenacity. These were sufficient to have deterred me from trying this practice, had I not known, from former experience, that many of them were ill founded.

"The state of the pulse is a most fallacious guide in the practice of venesection. A strong full pulse, accompanied with pain, in some particular part of the body, certainly indicates bleeding; but it may often be of service, when the pulse is in the opposite extreme.

"The fear of inducing dropsy, by the too free use of the lancet, is in most instances groundless. On the contrary, there are many diseases, diseases, accompanied with dropsy, particularly with œdema, where venesection is of the highest service. To such as have never seen, or tried, the practice, this account will appear incredible. I have not often bled in ascites, because this is frequently brought on, or accompanied, with some incurable organic affection; but in recent cases of anasarca, I have seen as good effects from venesection, as from any other practice.

"Fears, arising from the rotten discomposed state of the blood, are equally ill founded. Nothing can be a stronger proof of this than the present case. The blood was pretty much the same, as is generally met with in Diabetes, and seemed to agree with the description given by Drs. Dobson and Rollo. Little change took place in the first three bleedings. The fourth, however, was greatly altered. It had become sizy on the top; and, on cooling, the crassamentum acquired a considerable degree of firmness. The fifth was remarkably inflamed; the buffy coat was thick, firm, and contracted, to the size of a shilling. The coagulum had assumed a globular form, and become so tenacious, that it could be held out upon the point of a probe. The sixth was still firmer; and, in addition to former appearances, the serum had acquired a white, milky, or chylous appearance. These changes in the blood were singular and unexpected; but I have seen them often since. I remarked too that the veins, which, as the patient himself observed, were at first smaller than usual, became more and more turgid, and the blood flowed with greater force every successive bleeding.

"The effects of this practice on the general system were no less remarkable than on the blood. Even the first bleeding produced a degree of hilarity, to which for many weeks he had been a total stranger. After the third, the pristine vigour of his mind was completely restored, and his feelings rendered more comfortable. After the fourth, the pulse rose to about 80; became firm and regular, and some degree of perspiration appeared on different parts of the body. Still, however, there was no very remarkable change produced on the urinary discharge. After the fourth, there was evidently a relapse. The fifth seemed to act like a charm. The recovery after this was instantaneous and striking. The painful sensations in his bowels left him; the powers of virility returned; the gums became sound; the skin soft and perspirable; the saliva, the urine, and the alvine discharge, natural; in six days he returned to his work; in two months he was restored to his original strength."

Besides venesection, the patient took the carbonated ammonia, on account of an acidity in his stomach; and also to see what effect might be produced by furnishing the constitution with a substance which it usually secretes; but he seems to have derived little benefit from it. Antimonial powder taken till it induced vomiting, gave a temporary alleviation; but this is imputed more to the lessened quantity of the ingesta, than to any altered action in the seat of the disease. To lime-water, the author is willing to

 allow allow more efficacy; but observes, that in the present instance it was not tried till the cure was nearly completed.

The third case is more interesting, inasmuch as the subject was in that rank in life, of that disposition, that every means could be procured; and there is no reason to doubt the attention paid to them. Besides this, the author availed himself of the situation of his patient, to procure the occasional attendance of a physician, by whose correspondence he is relieved from every suspicion, which, however improbable, he might apprehend from the eagerness of his own discovery.—The patient was a student at Glasgow, twenty-three years of age, and of a constitution, which had always required considerable exercise to maintain health. The first invasion and future progress of the disease, is accurately discriminated by the author, with a minuteness highly creditable to himself, and not less useful to the reader. We must, however, content ourselves with some general remarks; and shall avail ourselves of the summary observations presented in the work, and which we cannot improve.

One of the early symptoms was a distressing pain, rather like lassitude than inflammation, across the regions of the kidnies. It was, however, severe enough to render the touch painful, and sometimes to render him almost unable to walk. Frequent and painful micturition followed; but the quantity of urine was never sufficient to excite alarm, had not its properties been altered. Pain about the corpora cavernosa penis at times, so considerable as to resemble tic doloreux, but without any alteration in the appearance. Acidity of stomach, which was in some measure constitutional, was much increased; a peculiar dejection of spirits, which is remarked in all the other subjects, and even traced in many cases related by others. This is all we need add to the author's own summary, which we shall now offer.

" A case is useful, in proportion to the unity of the disease, and the simplicity of the treatment. The obstinacy of the present instance, rendered the practice more complex and protracted than could have been wished; though nothing could be more decisive than the latter part of it. The patient, in a very few weeks, was restored, from the greatest distress and weakness, to the most perfect health. It becomes, therefore, an object of importance, to analyse the case; and, if possible, to separate the parts which are useful from the rest, and particularly from those which actually did harm. The time may be divided into different periods.

" During the first, which extended from the beginning of October till the 13th, the patient abstained from animal food, wine, spirits, and every thing of a heating or irritating nature. The bowels were kept moderately open; diluents were freely used, and an opiate taken at bed-time. This practice was persisted in, for eleven or twelve days, without any advantage. On the contrary, the number of times of voiding urine had increased, from eighteen to twenty-six, or twenty-seven. The pulse had become weak and intermitting intermitting; he had lost three pounds of his weight, and his strength and spirits were declining rapidly. The quantity of urine varied from five to eight pints; at times it equalled the whole liquid ingesta, and afforded, by evaporation, a copious extract.

"In the second period, which extends from the 13th October till the 26th of the same month, the patient, as to meat and drink, was allowed to live pretty much in his own way; the bowels were kept moderately open, and the opiate was occasionally continued at bed-time. On the evening of the 15th, and morning of the 16th, the patient was persuaded to eat beef steaks, and found all the symptoms greatly aggravated. The four succeeding days he was very abstemious, and every thing seemed to wear a more favourable aspect. On the 20th his appetite became keen, and a second indulgence produced a second relapse. On the 21st, the emetic had a good effect; but on the 23d his appetite returned, and it was thought hard to deprive a person of nourishment, when his body was sinking so rapidly, and when the depletion had been so considerable. He was bled six times, and had lost above an hundred ounces of blood. But, notwithstanding the continuance of some of the most distressing symptoms, the improvement, in this period, was very conspicuous. The pulse, from being weak and intermitting, had become firm and regular; the inflammation of the eyes was gone, and his vision had become strong and distinct; the blood, which he used to pass with his urine, had left him, and the urine itself was diminished in quantity, and more natural in its appearance. But the most striking improvement was the restoration of the mind to its wonted energy.

"In the third period, from the 27th October till the 12th November, his health and strength were rapidly on the decline. Various injections were tried to allay local irritation, but without any advantage. The perinæum was blistered repeatedly. At first, this seemed to afford some relief; but after a more extensive trial, it was found unavailing. The anodyne was increased from thirty-five to sixty drops, and afterwards to an hundred; but even this very large dose produced but a transient respite from the pressure. In this period, the greater part of his diet was animal food; for the last seven days it was entirely so; but made no impression on the disease.

"The fourth period, extends from the 12th November till the middle of December. In this interval the treatment was completely changed, and the progress towards recovery was uncommonly rapid. At the commencement, though the patient was greatly worse on the whole than he had been at all, yet, in some respects, he had not relapsed to his original condition. The mind continued serene, and even retained a considerable portion of energy; the blood was still inflamed, and of due consistence. In this period he was bled four times, and lost about fifty ounces of blood. Seven blisters, each of them nearly the size of the hand, were applied to the region of the kidnies, and a pretty rigid abstinence was observed. No medicine whatever was used till the 29th November, when he began the lime water, which seemed to have a happy effect on the state of his stomach and bowels, and probably contributed to his final recovery.

"From the above statement, some important inquiries arise. What share had the first six bleedings in accomplishing the cure? If the treatment, which was begun upon the 12th November, had been tried on the 1st October, would the result have been equally decisive? Are the three different branches of treatment, venesection, blistering, and abstinence, of equal importance? In what circumstances may any one of them supply the place of another, or be dispensed with altogether? Should the blood-letting be carried to a great extent at once, or will it answer the purpose better, to take it in smaller quantities, and at stated intervals? What are the criteria, which mark its being carried a sufficient length?"

We know not whether we should call the 4th case Diabetes or not. The patient had several symptoms of chronic indisposition; among the rest, unnatural appetite, frequent micturition, with no constant or considerable increase in quantity; a constant itching about the meatus urinarius, loss of strength, pain about the region of the kidnies, perpetual anxiety of mind, without sufficient cause. These, and some other symptoms, more particularly the hard dry inelastic skin, induced the author to remark the striking resemblance between the beginning of fever and the general symptoms of Diabetes; the principal difference between which, he remarks is, that the latter may continue for any indefinite length of time. We cannot, however, help remarking, that many of the symptoms alluded to, are common under many other indispositions; but that striking diagnostics attend each disease, and by those they are to be marked. In fever, we never meet with an inordinate appetite, nor that particular state of the urine which attends Diabetes. But if we even suppose that this case was not true Diabetes, it is highly worth recording; as one which shows with how much safety and advantage all the anti-phlogistic remedies may be used, in cases of extreme debility and mental depression.

The fifth was more rapid in its progress than we usually find Diabetes; and the conversion of the disease into diarrhœa, soon proved fatal. The observations annexed to this history show much learning, but are too logical. In pathological reasonings, the exceptions are so numerous, that they require more attention than the analogies. If the latter form the principal arguments, it is not difficult to confound all diseases and remedies also. We trust our author will receive this hint as it is meant; and, in a future edition of his work anticipate, in his own mind, many objections, of which a greater severity of criticism might make a very unfair advantage.

A long chapter on Practice follows. This abounds with good things, but is much too diffuse. We extract the following passage, not as the best by any means; perhaps, abstracted as it stands, it may may prove the worst. We acknowledge our disappointment, when men of real abilities, who possess discriminating powers with boldness of conception, should indulge the latter so far, as to expose their crudities unnecessarily. Many, which at first seem to shock the understanding, are made intelligible, appear plausible, and even rational, by being so far matured, that their true form may show itself divested of a drapery, both cumbrous and unnecessary.

"The buffy coat," says our author, when speaking of venesection, "has been a source of much error in the practice of venesection. Its absence in the first or second bleeding, and the patient's experiencing no relief from the operation, have often prevented the repetition of it, in the more advanced stages, when the buffy coat was sure to be found, and where bleeding was the only thing which could have saved the patient's life.

"There are many circumstances, with regard to this phenomenon, which cannot be easily accounted for. Its quantity has been supposed to point out the extent of disease, and the degree of danger. But it might with more propriety be considered, as pointing out the stage of recovery. It frequently does not exist, when the patient is at the worst; and is often in the greatest perfection, when the patient's health is so far recovered as to stand in need of no treatment.

"The globules of blood are so uniform in their appearance and texture, that we cannot easily suppose them to be destitute of organization. This may be admitted, without the necessity of supporting all the notions, which have been advanced concerning the vitality of the blood. Whether their organization be of the animal or vegetable kind, or something peculiar to themselves, they must have periods of growth, maturity, and decay. If they resemble organized bodies, with which we are acquainted, in embryo, they must be nearly transparent, and gradually acquire their natural colour, as they come to maturity. This is in some measure proved, by the circumstance, that the embryo in utero is colourless, though it must contain blood, and that blood must circulate through its body.

"From some reasonings of this kind, I have been led to suppose, that the lymph of the blood is the globules in their infant state; and that the buffy coat is a greater than ordinary proportion of lymph. This hypothesis is rendered probable by a number of circumstances, and is in some measure proved by the following fact. If the transparent buffy coat of inflamed blood be exposed to oxygen gas, or even to atmospheric air, it acquires a red colour. It does this more readily in the latter than in the earlier stages of reaction.

"The blood may consist of a promiscuous mass of globules, some of them in growth, others in decay. By disease, a number may be destroyed or rendered unserviceable: the carrying of these out of the system, and the rearing of new ones to supply their

 place, place, may be the object of reaction: the time and means necessary to accomplish that end, will necessarily modify the duration of the complaint."

By the globules, we suppose the author means the red globules. We should be glad to know, what is the difference of life and organization in a living body. If we admit organization, we admit not only life, but certain powers for self-preservation, or for some purposes in the animal, or for both; for to what other end can organization exist. If the buffy coat is sure to be found when the disease is so far advanced that reaction is powerful, can there be a more simple mode of accounting for it than Mr. Hunter's, viz. that the same increased action has taken place in the blood, by which its parts are rapidly separated; and in that separation, those which unite by what is called coagulation, form a firmer union, in proportion as they act with more strength. We are not certain whether we perfectly understand our author, that the buffy coat of inflamed blood will acquire a red colour when exposed to oxygen air. It has never occurred to us to make the trial; but it is certain that red particles will acquire the crimson tinge through the serum, though the buff never seems to change under the same situation. As we before remarked, we have extracted this as an objectionable passage; others might have been treated in the same manner; but still we are ready to admit, that few readers will peruse the practical remarks without advantage. We are, however, obliged to enter our protest against the indiscriminate and random use of mercury, which certainly did not at this time want Mr. Watts's sanction. However he may find it in the colder regions, it is certainly a remedy not to be given, without a certain determinate object in the warmer regions, and even in the English metropolis.

A chapter follows, giving an admirable account of one species of asthma, of which our author having seen several instances, reasons upon it as the general history of that disease. The remarks which follow are highly judicious, but the theory is, as may be expected, unsatisfactory. In this, the only blame we attach to our author, is in his attempting too much. That most diseases are preceded by chilliness, approaching to rigor, in proportion as the subsequent paroxysm is more or less severe, cannot be considered as a discovery; but the accuracy with which the succession of phenomena is traced, does credit to the author; particularly one circumstance too often over looked, but admirably described by Sydenham in his History of the Gout. We mean, that relief from all the threatening symptoms, and that temporary exhiliration which so frequently precede the immediate attack. A short chapter on cholera follows, and a longer one on colic. A chapter on consumption succeeds, introduced by the following paragraphs.

"In some instances, reaction scarcely takes place at all, and the patient dies soon after the application of cold. This has happened to people going into the cold bath, after they had been much heated. In other instances reaction comes on at an indefinite nite nite period, generally within one or two weeks, and terminates in fever, or in one or other of the complaints mentioned in last section. If the paroxysm be vigorous, and properly managed, a crisis obtains and the patient has a speedy and complete recovery. If reaction be feeble, a slow fever continues, which is aggravated by the slightest variation of regimen, exercise, or temperature. In other instances, after the balance is lost, there is almost no attempt in the system to recover itself: the patients take their food as well, or better, than in health; but they lose strength and spirits daily: the excretions are irregular, generally one of them in excess, and the rest deficient. But what chiefly characterises this state of body, is cold in the extremities, a general chilliness through the body, and a constant wish to sit near the fire: the patients too, from being social, cheerful, and enterprising, become dull, peevish, and partial to solitude.

"The perpetual low fever, and this want of tone in the system lead to fatal diseases; the first almost invariably to phthisis: the last to phthisis, scrophula, diabetes, dropsy, chorea, palsy, hydrocephalus, chlorosis, mania, &c. In almost every one of these a previous indisposition of weeks, months, and sometimes even years, may be distinctly traced; and very often, the first cause of the disease can be discovered."

Three cases follow, of which we shall only say that we have not met with similar ones. This may however be accounted for, from our not having witnessed an exactly similar treatment. The cases are certainly important, and the general success attending the practice, entitles them to a careful perusal.

A case of chorea follows, in a boy four years and 3 months old. Purgatives were insufficient to relieve him. At first only ℥ix. of blood were taken from the jugular vein, nine days after ℥x. more were taken, from which time he gradually recovered by the assistance of purgative remedies. The following are our author's remarks on the case.

"In two weeks after this, the patient was in perfect health, and has continued well ever since. I have had no opportunity of repeating the practice; but in obstinate cases, which do not yield readily to purgatives, venesection might, perhaps, be employed with advantage. This case seems to prove, that the convulsive motions do not depend on obstruction in the bowels, for they were completely removed, even while the hardened scybala remained. I am apt to think that the convulsive motions, the torpid intestines, the chilly state of body, and the peevish irritability of mind are merely symptoms of a diseased habit. The bleeding had an instantaneous effect in relieving them; after the system was repaired, a few grains of calomel enabled the intestines to discharge scybala, on which, doses three or four times as large had no effect in the earlier part of the treatment. The relief obtained at the time the scybala are discharged, is rather an incident, than the effect of the latter."

To

To this we shall make no other remark, but that we have usually found such diseases yield to chalybeates, sometimes after brisk purgatives, and sometimes without the use of them; but in saying this it is far from our intention to undervalue the author's practice, the success of which is a sufficient recommendation.

The work concludes with three cases of plethora, the last of which, after the bold practice of the author, was not relieved till by sudden menorrhagia the patient lost much more blood than all that had been taken at different periods by the lancet.

"I have ranked these three cases under the term plethora, because there was an obvious fulness of the sanguiferous system, and because a cure was effected by a profuse evacuation from that quarter. In the first instance the patient had a complication of diseases. The most urgent was the hysteric paroxysm, but she had also been long subject to menorrhagia and leucorrhœa. In the second instance there was a considerable tendency to an affection of the brain, at the time I saw him; but previous to that the hæmorrhoidal discharge and a debilitated state of the nervous system were the most conspicuous of his complaints; various means of relief had been tried in vain. In the last instance the lungs were in imminent danger; but it is probable she would have died of dropsy, in one form or other; it had commenced in the feet, and she had also symptoms of hydrothorax. She had an urgent thirst, and the urine was scanty and high coloured. In this case, it is doubtful, if depletion would have been carried a sufficient length to obtain a crisis, had not nature stepped in and taken the business into her own hands.

"In the treatment of all of them, my sole object was to restore the balance in the system, without regarding the local affections. After that was accomplished, not only the more conspicuous, but all the symptoms which marked a diseased habit disappeared."

Such is the nature of the work before us, of which, as far as our limits would permit, we have given we trust, an impartial, if not a perspicuous account. We discover many marks of genius in the author, but we suspect when he writes again he will do it with more caution. Yet we are pleased with his boldness, and ready to give him credit for a success, by which only he could have acquired it. Besides some transient remarks, which he will understand, we cannot help wishing he would in future, pay more attention to a very useful observation of his own.

"I believe, says he, that nothing has retarded the progress of medicine more than the attempts which have been made at Nosology. If half the pains had been taken to mark the slighter deviations from health, it would have led to results of greater importance; but these are often totally disregarded, and unless a regular combination of symptoms be discovered, so as to characterise a disease, the practitioner believes, and declares to the patient, that his feelings mislead him, and that he is really labouring under no complaint; unless it be a disordered imagination."

Not

Not only do we object to Nosology as a science, but in as much as it has been the means of introducing an affectation of language which is unbecoming the dignity of an useful art, or of a writer whose only wish is to inform. To what purpose are introduced the words *epistaxis*, *bulimia*, *morbus coxarius*, and some others we could mention. It is probable, that such may be the current language of the medical schools in the North; but this is not the case in the South. Though some of us are Greek scholars, yet etymology is not sufficient at once to present the meaning of such words; and if we consult nosological books, we find not merely the symptom intended to be designated, but an enumeration of others, which together form a *genus morborum*. These however are trifling objections in a work which contains so much original matter, that we cannot help recommending it to the reader.

---

*A Letter to the* Right Honourable SPENCER PERCEVAL, Chancellor of the Exchequer, &c. &c. &c., *on the Expediency and Propriety of regulating by Parliamentary Authority the Practice of Variolous Inoculation, with a View to the Extermination of the Small Pox.* By Sir EDM. CARRINGTON, late Chief Justice of the Island of Ceylon. 8vo. Lond. 1807.

THE learned author of this well penned address begins by expressing his reluctance at interrupting the Chancellor of the Exchequer from the important concerns of his office, but thinks, with much justice, that few things can be more interesting to the community than the present Inquiry. He then expresses a benevolent exultation on the Report of the Royal College of Physicians in London; to which, after it compliments for the simplicity of its style, and accuracy of its discrimination, he calls the particular attention of Mr. Perceval to the following passage

"Were encouragement given to vaccination, by offering it to the poorer classes without expence, there is little doubt but it would in time supersede the inoculation for the small-pox, and thereby various sources of variolous infection would be cut off: but till vaccination becomes general, it will be impossible to prevent the constant recurrence of the natural small-pox by means of those who are inoculated, except it should appear proper to the legislature to adopt, in its wisdom, some measure by which those who still, from terror or prejudice, prefer the small-pox to the vaccine disease, may, in thus consulting the gratification of their own feelings, be prevented from doing mischief to their neighbours."

This passage furnishes a text for the author to retrace and comment on our ancient laws, "which dignified the ages of our ancestors, and which, instead of gratefully applauding as wise, we so frequently pity as barbarous."

Respecting the first proposal of offering the benefit of gratuitous inoculation to the poor, we know not how it can be done with more attention than for some time past in the metropolis. There are four public charities instituted for that purpose, and the public is every where respectfully informed of the different stations in many other other parts of the town. We sincerely hope the same facility may be established in the country.

The author goes on to remark the evils of variolous inoculation. It cannot be wondered at if he offers little new on a subject which has been so long agitated. We shall therefore confine ourselves to the legal part of the pamphlet, as that appears to come more immediately within the scope of the author's inquiries.

In order to reconcile that part of the public whose prejudices might be startled from the apprehension of an encroachment on English liberty, we are reminded, that in the year 1720, when projectors were so numerous as to give a dangerous turn to the activity of the crafty, the legislature thought it necessary to interfere. That long before that time they had erected lazar-houses for the reception of those afflicted with elephantiasis, a disease supposed to have been introduced shortly after the Norman conquest. A statute, *de leproso amovendo*, is quoted at some length, and a quotation from Coke shows, that even that illustrious lawyer did not think such an act contrary to liberty. Among the various cases in which a man may, by the law of the land, be imprisoned by force of the King's writ, he mentions, for saving the people from contagion or infection. The same commentator afterwards confirms this opinion, in recognizing the true sense of certain doctrines of pleading. Bacton and Fleta lay it down as a rule, that a person who by law would be considered, either from contagion, or the deformity of appearance, unfit to be seen abroad, may claim excuse from appearing to plead.

The next visitation of epidemic diseases, the author considers the plague, of which he supposes the sweating sickness a mere species. If he is mistaken in this last suggestion, fair allowances must be made for the particular line of his studies, which are not medical.

On the subject of plague, it is remarkable that till the first year of James I. no provision had been made for preventing the extension of that disease. A proclamation had indeed been made in the 24th of Edward III. as cited by Rymer, which sufficiently demonstrates the ravages that disease had committed, by the attempts made to provide against them.

The statute of James, after a preamble reciting the ill conduct of some of the diseased, enacts, "That if any person or persons infected, or being or dwelling in any house infected, shall be by the mayor, bailiffs, constable, or other head officer of any city, borough, town corporate, privileged place, or market town, or by any justice of peace, constable, headborough, or other officer of the county (if any such infection be out of any city, borough, town corporate, privileged place, or market town), commanded or appointed, as aforesaid, to keep his or their house, for avoiding of further infection, and shall, notwithstanding, wilfully and contemptuously disobey such direction and appointment, offering and attempting to break out and go abroad, and to resist, or going abroad, and resisting such keepers and watchmen as shall be appointed, as aforesaid, to see them kept in; that then it shall be lawful for such watchmen with with violence to enforce them to keep their houses; and if any hurt come by such enforcement to such disobedient persons, that then the said keepers, watchmen, and any other their assistants, shall not be impeached therefore: (2) and if any infected person, as aforesaid, so commanded to keep house, shall, contrary to such commandment, wilfully and contemptuously go abroad, and shall converse in company, having any infectious sore upon him uncured, that then such person and persons shall be taken, deemed and adjudged as a felon, and to suffer pains of death as in case of felony: (3) but if such person shall not have any such sore found about him, then for his said offence to be punished as a vagabond in all respects should or ought to be, by the statute made in the nine and thirtieth year of the reign of our late sovereign lady Queen Elizabeth, for the punishment of rogues and vagabonds; and further, to be bound to his or their good behaviour for one whole year."

We have transcribed this act from the author, who remarks, that the same, with such modifications as the present state of society requires, may be applied with great public benefit to the regulation of variolous inoculation.

The author next remarks, that though a period of 132 years has now elapsed since the appearance of the plague in this nation, yet nothing has lessened the vigilance of government over the health of the people. No fewer than eight several Acts of Parliament have from time to time been framed to regulate quarantines. That of 38th of his Majesty provides not only against the plague, but all other contagious diseases which have or may rage in foreign parts. These acts have since been repealed, and their contents consolidated in the 45th of his Majesty, cap. 10. By this act, all vessels are to perform quarantine coming from, or having touched at any place, where his Majesty, by advice of his privy council, shall have declared it probable that the plague, or any other infectious disease or distemper, may be brought, authorising them to cut off all communication between any person infected with such disease, and the rest of his Majesty's subjects.

The wisdom of these provisions, it is added, has never been questioned. "But," continues our author, "will it be wise or consistent while we thus guard our country from exterior infection, voluntarily to cherish an infection equally malignant within its bosom?" With this view he proposes, that if variolous inoculation be not entirely prohibited, at least that it should be immediately regulated and limited. We have been thus particular on the contents of this pamphlet, not because we ever entertained a doubt of the constitutional power of the legislature to make laws for the preserving the health of the subject, but to satisfy those few who may retain such doubts. We perfectly agree too, with the learned author, that "a committee of the House of Commons, with the assistance they may command, will be the best judges of the practicability, the expediency, the necessity, of these or similar provisions." To this tribunal we commit the inquiry, not doubting that the period elapsed since since it has been instituted, will be sufficient to induce that maturity of judgment which so serious a question demands.

---

*Important Researches upon the Existence, Nature, and Communication of the Venereal Infection in Pregnant Women, New-born Infants, and Nurses. By the late* P. A. O. Mahon, Chief Physician to the Venereal Hospital du Vaugirard, &c. &c. &c. at Paris. *These are Contrasted with the new Opinions of the late* John Hunter *upon this Subject, together with Observations thereon.* By Jesse Foot, Surgeon. 8vo. Lond. 1808.

Our respect for the character whose opinions are here examined might have subjected us to the suspicion of partiality, had we gone minutely into these "Researches." We should however have endeavoured to do justice to both parties, could we have understood the author. But either he, the translator, or printer, has so obscured several passages, as to render us doubtful concerning the rest.

"Chapter I.—*Upon what has been the Opinion of Physicians on the Venereal Disease in New-born Infants?*

"In order to discuss these researches with the greatest perspicuity, I shall arrange the different authors who have written on the venereal disease in children, into two classes, and shall take for the basis of that division, not the disposition of the times when these authors lived, but the importance and extent of their works. In the first class will be comprehended the principal authors who have written since the beginning of the fifteenth century, until the middle of the eighteenth century. The second class will comprehend those who have written since the year 1750, until this time.

"Article I.—*The principal Authors who have written since the Commencement of the Fifteenth Century, until the Middle of the Eighteenth Century.*

"In surveying the most compleat collection of ancient authors who have written on the venereal disease, Aphrodisiacus de Luisinus published for the first time in 1506." *

In the mode of dividing these two classes, we suppose there must be some obscurity in the author, which by the note, it appears the translator has endeavoured to rectify in part, but we still remain in the same uncertainty. By Aphrodisiacus de Luisinus, we conceive must be meant the collection of Aloysius Luisinus, to which he gave the name of Aphrodisiacus.

We shall offer one more passage to convince the reader of the difficulty of knowing Mr. Hunter's meaning, if we are to take it from Dr. Mahon.

"Mr John Hunter has adopted a decided proposition, that pus alone contained the venereal virus, and that all cases that were venereal

---

* This is surely the commencement of the sixteenth century. J. F.

venereal that did furnish pus, except those of an incipient chancre or a gonorrhæa, could not communicate the disease. What ought to be concluded from such propositions, but entirely new and extraordinary consequences, and which appear astonishing to those to whom those principles have not the same degree of certainty and evidence. We will not examine here what erroneous consequences upon the nature and treatment of the venereal disease might be construed from the two propositions of which Mr. Hunter thought to prove the fact; we will content ourselves with analysing those arguments upon which he rested, to convince the want of communicability of the venereal virus from children to their nurses, and from the nurses to their children. We will hear Mr. John Hunter's opinion."

The following is the beginning of Dr. Mahon's remark on some of the cases produced by Mr. Hunter, of diseases resembling lues, but which, after stating minutely, he conceives are not venereal.

"The observations that are given by Mr. John Hunter, as diseases resembling the venereal disease, but on which he is mistaken in taking them for it."

"Dr. Mahon.—Critical remarks upon these observations, which prove that these diseases were true affections of the venereal disease."

Now, if they really were the venereal disease, how could Mr. Hunter be mistaken in taking them for it. However, in perusing Mr. Hunter's account, it appears that he believes they were not venereal. But to proceed with Dr. Mahon.

"We shall see that women infected with the venereal disease are subject to miscarriages, and that the waters are of a bad species, and the children born are robbed of the epidermis.

"The vessicles are what we have termed *phlictènes* in the table of symptoms. We say this symptom is frequent, and we shall place it among the unequivocal signs of the inoculation of the venereal disease."

We refer those who are satisfied with these unequivocal signs to the "Researches," or the Translation.

